# Modified nutrient management protocol for optimum biomass production, nutritional quality, and flavor-related phytochemical properties of hydroponic-grown kale (*Brassica oleracea*)

**DOI:** 10.3389/fpls.2025.1629432

**Published:** 2025-08-29

**Authors:** Teng Yang, Uttara Samarakoon, James Altland

**Affiliations:** ^1^ Agricultural Technical Institute, The Ohio State University, Wooster, OH, United States; ^2^ United States Department of Agriculture Agricultural Research Service, Wooster, OH, United States

**Keywords:** calcium nitrate, hydroponics, nitrate accumulation, nitrogen supplementation, nutrient imbalance, nutrient management

## Abstract

**Introduction:**

Nutrient supply in hydroponic leafy green production is often not aligned with crop-specific requirements. Kale (*Brassica oleracea* ‘Red Russian’) has been shown to exhibit higher nitrogen (N) demand than other leafy greens. Conventional nutrient management relies on a two-part water-soluble fertilizer system—Part A with macronutrients and micronutrients and Part B with calcium nitrate (Ca(NO_3_)_2_)—to maintain electrical conductivity (EC), but this approach may not optimize N supplementation or crop quality.

**Methods:**

We evaluated a modified protocol in which only Ca(NO_3_)_2_ was supplied during the final production week, replacing the standard two-part adjustment. Plant biomass, nutrient composition, phytochemicals, and physiological traits of hydroponically grown kale were assessed.

**Results:**

The Ca(NO_3_)_2_-only treatment significantly increased shoot biomass, shoot-to-root ratio, and uptake of N, calcium, and magnesium by 28.5%, 22.1%, 46.0%, 27.5%, and 14.4%, respectively, compared with conventional management, suggesting N and calcium were key limiting factors for shoot growth. Nitrate accumulation in shoots also increased but remained within safe consumption limits. Phytochemical analysis revealed reductions in anthocyanins and vitamin C, alongside a slight increase in glucosinolates. No significant changes were observed in photosynthetic traits, root growth, or water and acid use.

**Discussion:**

Targeted N supplementation with Ca(NO_3_)_2_ enhanced growth and nutrient uptake in kale but introduced tradeoffs in phytochemical composition. These results underscore the potential of crop-specific nutrient strategies to improve both yield and nutritional quality of hydroponic leafy greens in controlled environment systems.

## 1 Introduction

Kale (*Brassica oleracea* var. acephala) is a non-heading, leafy green vegetable that has been cultivated for over 6,000 years, with its origins tracing back to the eastern Mediterranean region (Maggioni et al., 2018). As a member of the *Brassica oleracea* species, which includes broccoli (var. italica), cabbage (var. capitata), cauliflower (var. botrytis), collard greens (var. viridis), Brussels sprout (var. gemmifera), savoy (var. sabauda), and kohlrabi (Gongylodes Group), kale exhibits extensive genetic diversity, resulting in variations in morphology, color, and nutrient composition (Miao et al., 2020). Due to its adaptability and high cold tolerance, kale can be cultivated across diverse climates, thriving in both traditional open-field systems and controlled environment agriculture (CEA) settings (Kim and Chung, 2018; Low, 2019). Kale is widely recognized for its dense nutritional profile and associated health benefits. It is rich in bioactive compounds such as carotenoids, flavonoids, and glucosinolates (Ferioli et al., 2013; Schmidt et al., 2010), which contribute to its strong antioxidant properties and potential protective effects against cardiovascular diseases and various cancers (Šamec et al., 2019). Additionally, kale serves as an excellent source of essential vitamins and minerals, including vitamin K, calcium, magnesium, and iron, with studies indicating that calcium absorption from kale surpasses that of milk (Heaney and Weaver, 1990). It is also one of the highest dietary sources of lutein and β-carotene, both of which are linked to a reduced risk of age-related eye diseases and other chronic conditions (Kopsell et al., 2004; Lefsrud et al, 2006).

Despite its growing popularity, research on kale remains limited compared to other *Brassica* vegetables such as broccoli and cabbage (Kim et al., 2017). Its nutritional quality is influenced by a range of factors, including cultivar type, environmental conditions (e.g., temperature, nutrition, and light exposure), and developmental stage (de Azevedo and Rodriguez-Amaya, 2005; Ku et al., 2016; Walsh et al., 2015). For instance, Yang et al. (2024) reported that several important health-beneficial compounds (particularly vitamin C, anthocyanin, and phenolic compounds) in kale leaves declined as electrical conductivity (EC) levels increased. However, important gaps remain in understanding the variability of phytonutrient content among kale cultivars and the effects of environmental conditions in both traditional and controlled production systems. Ahn et al. (2021) compared three nutrient dosing strategies in hydroponically grown *Brassica* species, concluding that EC-based nutrient management yielded more consistent and reproducible nutrient variations in the root zone. Expanding on this, Bosman et al. (2024) employed real-time pH and EC control through dynamic ammonium-to-nitrate ratio adjustments in an ebb-and-flow hydroponic system for *Brassica oleracea* var. acephala. While the approach proved effective, its complexity may limit practical scalability. Mao et al. (2022) identified optimal nitrogen and magnesium concentrations for Chinese kale (*Brassica albograbra* Bailey) at 105 ppm and 16.8 ppm, respectively, though the wide experimental ranges (35–315 ppm N and 6–50.4 ppm Mg) limit their utility for precise grower recommendations. Likewise, Martínez-Castillo et al. (2022) investigated the effects of electrical conductivity (0.5–2.0 dS·m^-^¹) on kale (*Brassica oleracea* cv. Dwarf Blue Curled Scotch) in a perlite-based hydroponic system; however, the applicability of their findings to liquid-based hydroponics remains uncertain due to differing root-zone dynamics and nutrient transport mechanisms.

Previous studies (Yang et al., 2024) demonstrated that although collard and kale belong to the same botanical family, they exhibited distinct responses to electrical conductivity (EC) in hydroponic systems. Collard achieved optimal growth performance, with a higher biomass accumulation, nutritional content and phytochemical levels under EC 1.8. In contrast, kale showed a positive correlation between increased EC levels and improved growth performance. Notably, even when EC was maintained at 2.1 mS·cm^−1^, total nitrogen depletion occurred in hydroponic systems toward the end of the production cycle, highlighting the necessity for optimized nutrient supplementation. This study examined the impact of enhanced nitrogen supplementation with calcium nitrate (Ca(NO_3_)_2_) while reducing the remaining macro and micronutrients during the final week of production, on biomass yield, nutrient uptake, and phytochemical composition of hydroponically grown kale.

## 2 Materials and methods

### 2.1 Plant material and growing conditions

**Figure 1:**
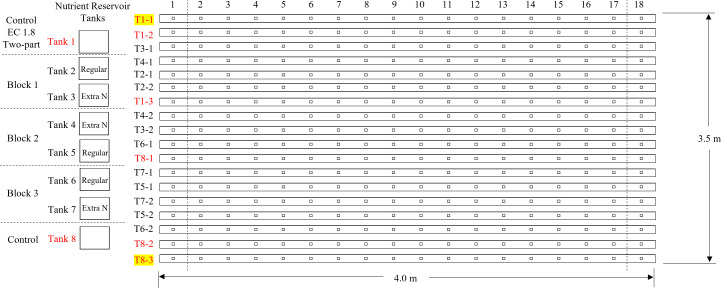
Growing channel layout showing the location of treatments replications. Channels 1–1 and 8–3 were closed as borders.

The experiments were conducted from March 28, 2022 to April 25, 2022 using a nutrient film technique (NFT) system (CropKing, Lodi, OH, USA) established in a double polyethylene-plastic covered greenhouse at the Ohio State University, Wooster Campus, Ohio (40.78° N, 81.93° W). Nutrient solutions were stored in eight ultra-violet stabilized plastic tanks. Each tank was randomly connected to four of 16 growing channels that were each 4 m long (**Figure 1**). Two border channels were closed. Each growing channel was 0.2 m apart with the capacity to grow 18 plants. Each plant grown within a channel was 0.2 m apart from each other. A galvanized steel frame was used for supporting the growing channels. Each reservoir tank was equipped with one high-efficiency circulation pump (Model 3WY90; Dayton Electric Mfg., Niles, IL, USA) to deliver nutrient solutions to the growing channel and drain the nutrient back to the reservoir tank.

**Figure 2:**
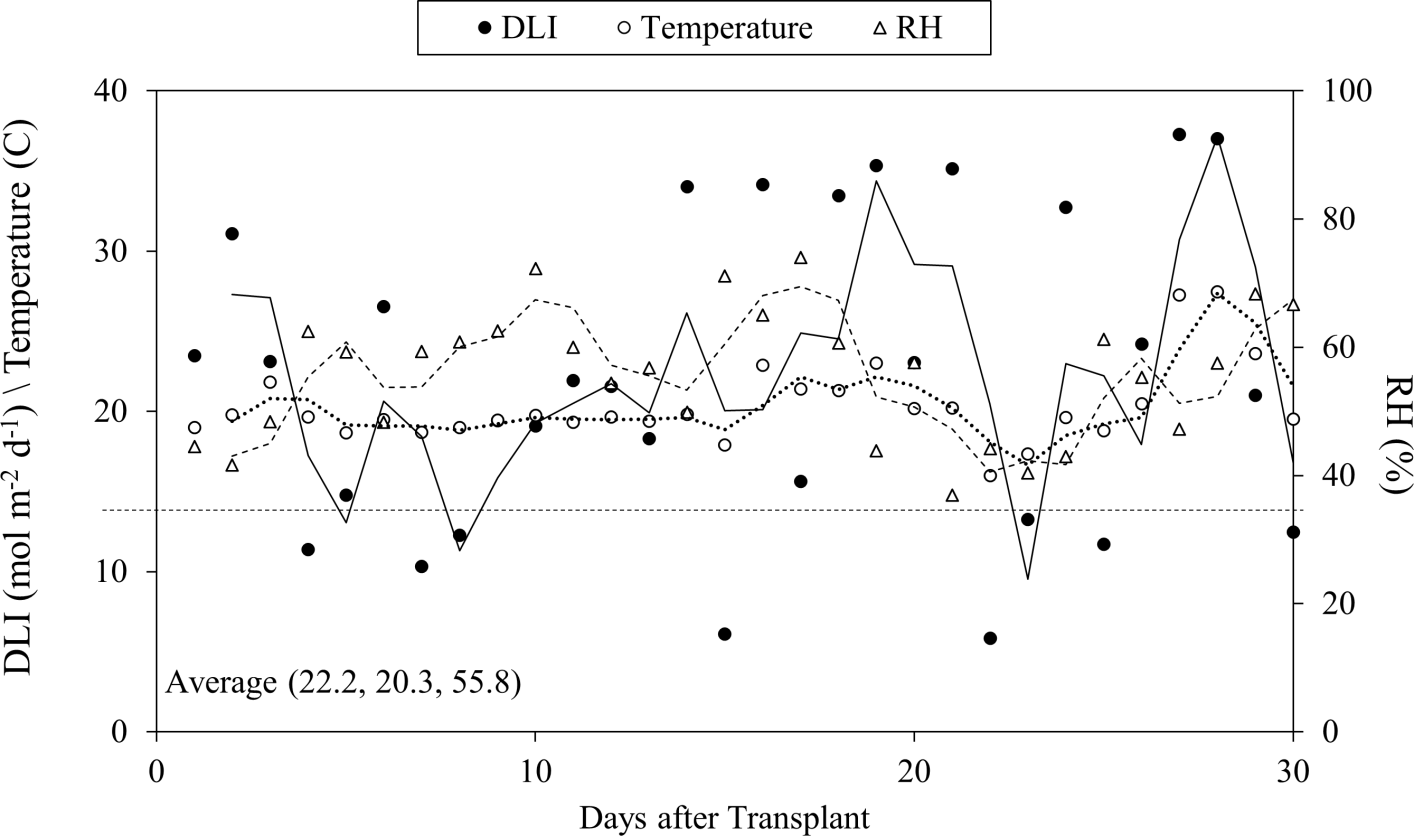
Growth environment of daily light intensity (DLI), air temperature, relative humidity (RH) in the greenhouse during the study.

The air temperature and air humidity of the greenhouse were measured every 10 s with a humidity and temperature probe (INTERCAP^®^ HMP50; Vaisala, Helsinki, Finland). Photosynthetic photon flux density (PPFD) was provided by natural light and high-intensity discharge (HID) lamps (400-Watt high-pressure sodium (HPS), Energy Technics Horticulture Lighting, York, PA, USA) for 16 h per day and measured every 10 s with a sun calibration quantum sensor (SQ-110-SS; Apogee Instruments, Logan, UT, USA). Air temperature, air humidity and average light intensity were logged every 10 s with a micrologger (CR3000; Campbell Scientific, Logan, UT, USA). During the experiment, the average (± standard error) day and night air temperature, air humidity and daily light integral of PPFD were 23.7± 0.2°C, 16.6 ± 0.05°C, 55.8 ± 1.9% and 22.2 ± 1.8 mol·m^−2^·d^−1^, respectively (**Figure 2**).

The experiment was conducted using Kale (*Brassica oleracea*) ‘Red Russian’ (Johnny’s Selected Seeds, Albion, ME, USA). A water-soluble fertilizer (Hydro Grow Leafy Green Fertilizer; 4.3% N-9.3% P-35% K: Crop King, Lodi, OH, USA) at 100 mL.L^−1^ and calcium nitrate (CropKing) at 78 mL.L^−1^ was used as fertilizer stock solution which were used to prepare feeding solutions in the study. Seed germination was done in 162-cell foam (Horticubes^®^; Smithers Oasis, Canton, OH, USA) using a propagation system for hydroponic crop production with nutrient circulation. A diluted solution with EC 1 to 1.2 dS·m^−1^ and pH 5.8 was used when first leaves appeared. Kale seedlings were transplanted in the 3rd week from germination. The time from transplanting to harvest was 4 weeks.

### 2.2 Treatments and experimental design

The nutrient concentrations in the initial feeding solution are shown in **Table 1**. Supplemental water (EC 0.8 dS·m^−1^, pH 6.4 and alkalinity 192.8 mg·L^−1^) was added into the reservoir tanks daily to maintain the water level, then EC 1.8 dS·m^−1^ and pH 5.8 were adjusted within ±0.05 range according to the treatment settings. EC and pH of the feeding solution were monitored using EC meter (COM-100, HM Digital Inc., Redondo Beach, CA, USA) and pH meter (PH-200, HM Digital Inc., Redondo Beach, CA, USA), respectively. The EC of the feeding solution was adjusted to 1.8 ± 0.05 dS·m^−1^ daily using the fertilizer stock solutions (increase EC) and water (reduce EC) during the first three weeks of the study. After the adjustment of EC, the pH of the feeding solution was adjusted to 5.8 ± 0.05 daily using 10% citric acid (reduce pH) or water (increase pH). In the final (fourth) week of the study, six reservoir tanks were grouped into three blocks (**Figure 1**), and the two reservoir tanks in each block were randomly assigned with one of the two treatments (**Table 2**): regular two-part management (adjust EC with both stock solutions of Hydro Grow Leafy Green Fertilizer and calcium nitrate until the end of production) and only calcium nitrate in the final week (adjust EC with only calcium nitrate during the 4th week of production).

**Table 1:** Macro-nutrient compositions and concentrations used in the nutrient solution with EC of 1.8 dS·cm^-1^ and pH of 5.8.

Parameter	Concentrations (mg.L^-1^)
Total nitrogen (N)	129.2
P	48.3
K	183.2
S	52.1
Ca	136.5
Mg	32.7

**Table 2:** Treatments (regular two-part management or only calcium nitrate in the final week) and experimental design in this study.

Treatment	Weeks 1–3	Week 4
Regular	Hydro Grow Leafy Green + Ca(NO_3_)_2_	Hydro Grow Leafy Green + Ca(NO_3_)_2_
Ca(NO_3_)_2_	Hydro Grow Leafy Green + Ca(NO_3_)_2_	Ca(NO_3_)_2_ only

Supplemental water was collected for measurements of EC, pH, redox, and alkalinity (CaCO_3_) using a titrator (T7; Mettler Toledo, Columbus, OH, USA) with an autosampler (InMotion Max; Mettler Toledo) and a pH probe (DGi115-SC; Mettler Toledo). The feeding solution from each reservoir tank was collected weekly for nutrient composition measurements. The nutrient composition of supplemental water and feeding solution was determined with ion chromatography systems (IC 600; Thermo Fisher Scientific, Waltham, MA). The total nitrogen (TN) and total organic carbon (TOC) concentrations of supplemental water and feeding solution was determined using the total organic carbon analyzer (TOC-LCSN, Shimadzu, Kyoto, Japan).

Each growing channel contained 18 kale seedlings. To avoid the edge effects, data were not collected from outermost growing channels and the two plants cultured on the edge within each growing channel. Thus, there were 6 replicate channels for each treatment and 16 plants in each channel. Treatments were assigned to each channel in a completely randomized design.

### 2.3 Measurement of gas exchange properties

Gas-exchange measurements were performed using a portable gas exchange system (LI-6400XT; LICOR Biosciences, Lincoln, NE) equipped with a 6-cm^2^ leaf chamber with built-in LEDs (470 and 665-nm peak wavelengths for blue and red LEDs, respectively). Illumination was supplied at a PPF of 1,000 μmol·m^−2^·s^−1^ by red and blue LEDs at a ratio of 9:1 under 20°C in the leaf chamber when supplemental lighting was in use. The reference CO_2_ concentration and flow rate through the chamber were 400 μmol·mol^−1^ and 500 μmol·s^−1^, respectively.

Four plants of each channel (24 plants for each treatment) were selected for the photosynthetic property measurement on the 1st, 2nd, 3rd and 4th weeks after transplant. The third fully expended youngest leaf was selected from each plant for the measurements. The measurements of photosynthetic rate (Pn), transpiration rate (Tr), and stomata conductance (Gs) were conducted between 9:00 am and 16:00 pm at a PPFD of 1,000 μmol·m^−2^·s^−1^. Readings were taken when the coefficient of variation (i.e., sample CO_2_, sample H_2_O, and flow rate) was less than or equal to 0.2% (stable), which typically occurred within 10 min. The intrinsic water use efficiency (WUE) was calculated by dividing Pn by Tr (Chaves et al., 2004).

### 2.4 Measurement of relative chlorophyll content and chlorophyll fluorescence

On the 1st, 2nd, 3rd and 4th week after transplant, four representative plant samples were selected from each growing channel (eight plants for each nutrient reservoir tank and 24 plants for each treatment) for relative chlorophyll content (SPAD; an index of chlorophyll content per unit leaf area) and chlorophyll fluorescence measurement. The SPAD readings were taken on each fully expanded leaf with a chlorophyll meter (SPAD-502, Minolta Corporation, Ltd., Osaka, Japan). Five readings per leaf were taken at the central point of a leaf between the midrib and the leaf margin and the values were averaged and recorded. Chlorophyll fluorescence was measured immediately after dark adaptation using a Plant Efficiency Analyzer, Handy PEA (Hansatech Instruments, King’s Lynn, England). The right dark period duration (20 minutes) and the optimal light intensity (3,500 µmol·m^−2^·s^−1^) were evaluated according to Rodolfi et al. (2021).

### 2.5 Harvesting and yield measurements

At four weeks after transplant, twelve kale plants per channel were harvested. Plants used for the photosynthetic property measurement were not included in the final harvest due to possible mechanical disruption of tissues. All plant samples were separated into roots and shoots and fresh weight was recorded. All shoot samples were collected for leaf area measurement. Each leaf sample was scanned for leaf area by using a portable laser leaf area meter (CI-202, CID Bio-Science, Inc., Camas, WA, USA), and recorded for the calculation of total leaf area. Then plant samples were oven-dried at 68°C until a constant weight was reached before taking the dry weight. All dried samples were filtered through a 10-mesh screen after grinding with a sample mill (Cyclotec™ 1093, FOSS Analytical, Denmark) and kept in plastic vials for tissue nutrient analysis.

### 2.6 Measurement of tissue nutrient analysis

Plant tissue nutrient analysis was conducted using leaf samples of kale (18 plants per treatment) at Ohio State University’s Service, Testing, and Research (STAR) laboratory (Wooster, OH, USA) to investigate the variations in nutrient uptake under two different treatments. Total concentrations of plant-essential elements (P, K, Ca, Mg, S, Al, B, Cu, Fe, Mn, Mo, Na, and Zn) were determined by microwave digestion with HNO_3_ followed by inductively coupled plasma (ICP) emission spectrometry according to Jones (Jones et al., 1991). Nitrate nitrogen in plant tissue samples were determined by the NO_3_-N cadmium reduction method (Gavlak et al., 2005). Total nitrogen in plant tissue samples was determined by the Dumas method according to Association of Official Analytical Chemists (AOAC, 1990).

### 2.7 Measurement of chlorophyll and carotenoids

Chlorophyll a, chlorophyll b and carotenoids were extracted from 25 mg fresh leaf tissues using 100% methanol as the solvent. Samples were kept in a dark room at 4°C for 24 h. Quantitative determination of total chlorophyll was carried out immediately after extraction. Absorbance readings were measured at 661.6 and 644.8 nm for chlorophyll pigments and 470 nm for total carotenoids. Chlorophyll and carotenoids concentrations were calculated by Equations 1–4 (Lichtenthaler, 1987):


(1) $$Chl_{a}=11.25A_{661.6}-2.04A_{644.8}$$

(2) $$Chl_{b}=20.13A_{644.8}-4.19A_{661.6}$$

(3) $$Chl_{a+b}=7.05A_{661.6}+18.09A_{644.8}$$

(4) $$Car_{x+c}=\frac{\left(11.24A_{470}-1.90Chl_{a}-63.14Chl_{b}\right)}{214}$$

### 2.8 Measurement of total anthocyanins

Total anthocyanins were estimated by a modified spectrophotometric method of Sukwattanasinit et al. (2007). Dried and ground plants samples weighing 160 mg were macerated with 0.1% HCl in 75% MeOH (25 mL) at room temperature in a dark room for 24 hours, and then filtered. Two 0.5 mL of sample extracts were separately mixed with 2.5 mL of KCl buffer (0.025 M, pH 1.0), and 2.5 mL of sodium acetate buffer (0.4 M, pH 4.5). After 30 minutes, the absorbance of each mixture solution was measured at 520 nm (A520) and 700 mn (A700) using a UV-Vis spectrophotometer (Genesys 180, Thermo Fisher Scientific Inc., Waltham, MA, USA). The absorbance of the measured solution (A) was calculated by the following equation:


$$A={\left(A_{520}-A_{700}\right)}_{pH1.0}-{\left(A_{520}-A_{700}\right)}_{pH4.5}$$

The anthocyanin concentration in the sample was calculated against the simulated calibration curve of methyl orange and expressed as percentage of anthocyanin concentration based on dried plant weight.

### 2.9 Measurement of total concentration of phenolic compounds

Total concentration of phenolic compounds was determined by the spectrophotometric method of Stratil et al. (2006). Fresh leaf samples were cut into small pieces (<0.5 cm) and lyophilized at -52°C for 48 to 72 h in a freeze dryer lyophilizer (Virtis Freezemobile 12SL, American Laboratory Trading, East Lyme, CT, USA). The dry matter was grinded using an automated mini tissue homogenizer (1600 MiniG, SPEX SamplePrep, LLC, Metuchen, NJ, USA). Then two portions of 500 mg of pulverized lyophilized plant tissue were weighed for acidic and non-acid extraction. The non-acidic sample was added with 10 mL of 1:1 methanol: water (purified to 18.2 MOhm.cm; Synergy Water Purification System, MilliporeSigma, Burlington, MA, USA), and the acidic sample was added with 10 mL of 1:1 methanol: 2.4 M HCl. Both samples were vortexed for 2 minutes, then incubated at 83°C in a water bath for 150 minutes, and vortexed for 20 seconds every 30 minutes during the incubation period. After incubation, 10 mL of pure methanol was added to each sample and vortexed for 20 seconds. Both samples were centrifuged at 6,000 rpm for 10 minutes, then the supernatants were neutralized with 5 M NaOH. Then 200 ul of sample supernatant was mixed with 400 ul of 10% Folin-Ciocalteu reagent and 1.6 ml of 700 mM Na_2_CO_3_ and incubated for 2 hours at room temperature. Absorbance readings were measured at 760 nm using a UV-Vis spectrophotometer. The total concentration of phenolic compounds in the sample was calculated against the standard curve of a gallic acid solution.

### 2.10 Measurement of vitamin C

Concentration of vitamin C was determined by the spectrophotometric method of Kapur et al. (2012). Fresh leaf tissues of 10 g were grinded using an automated mini tissue homogenizer (1600 MiniG, SPEX SamplePrep, LLC) with 25 mL phospho-acetic acid. The mixture was centrifuged at 4,000 rpm for 15 mins, then the supernatants were collected. The 5 mL supernatant sample was added with 50 uL of 3% bromine water, 25 uL of 10% thiourea, 1 ml of glacial acetic acid, and 1 mL of 2,4-DNPH (2 g of 2,4-dintrophenylhydrazine and 4 g thiourea dissolved in 100 mL of 4.5 M sulfuric acid), then placed into a water bath at 37°C for 3 hours. After incubation, 5 ml of chilled 85% H_2_SO_4_ was added, and immediately read for absorbance at 521 nm using a UV-Vis spectrophotometer. The total concentration of vitamin C in the sample was calculated against the standard curve of ascorbic acid solution.

### 2.11 Measurement of total glucosinolates

Total glucosinolates were estimated by spectrophotometric method of Mawlong et al. (2017). A fresh plant shoot sample of 10 g was homogenized in a 2 ml vial with 80% methanol. This homogenate was centrifuged at 3,000 rpm for 4 min after keeping overnight at room temperature. The supernatant was collected after centrifugation and made up to 2 ml with 80% methanol. The extraction of 100 μl was used for estimation. A volume of 0.3 ml double distilled water and 3 ml of 2 mM sodium tetrachloropalladate (58.8 mg Sodium tetrachloropalladate + 170 μl concentrated HCl + 100 ml double distilled water) were added to the sample. After incubation at room temperature for 1 h, absorbance was measured at 425 nm using a UV-Vis spectrophotometer (Genesys 180, Thermo Fisher Scientific Inc., Waltham, MA). A blank sample, prepared using the same procedure but without plant extract, was included as a control. Total glucosinolates were calculated by applying the absorbance value of each sample taken at 425 nm into the predicted formula:


$$y=1.40+118.86\times A_{425}$$

### 2.12 Statistical analysis

Data were analyzed using JMP^®^ for Windows, Version 17.0 Pro (SAS Institute Inc., Cary, NC). Statistical differences were determined using a one-way analysis of variance (ANOVA) followed by Tukey’s honestly significant difference (HSD) test (P = 0.05). In addition, multiple regression analysis with simple linear and non-linear models were conducted, and regression plots were presented in the figures to display the changes of plant response parameters during the crop cycle. The significance of regression coefficient was indicated with *, ** or *** at P of 0.05, 0.01 or 0.001, respectively.

## 3 Results

### 3.1 EC and pH adjustments and macro nutrient concentrations in the feeding solution

The amount of acid and water usage were similar, while the volume of stock solutions were significantly different between treatments (**Table 3**). Compared to the control, Ca(NO_3_)_2_-only treatment utilized 54.5% less stock solution and 78.0% more calcium nitrate stock solution in the final week.

**Table 3:** Daily usage of stock solutions, acids and water in the nutrient feeding solution to adjust EC to 1.8 dS·m^−1^ and pH to 5.8 during the study. Data represents the mean of 30 measurements take.

Treatment	Stock solution (mL)	Calcium nitrate stock solution (mL)	10% H_2_SO_4_ (mL)	H_2_O (L)
Regular two-part	24.4 a	24.4 b	4.8 a	4.3 a
Only Ca(NO_3_)_2_ in final week	11.1 b	43.4 a	5.3 a	3.9 a

**Figure 3:**
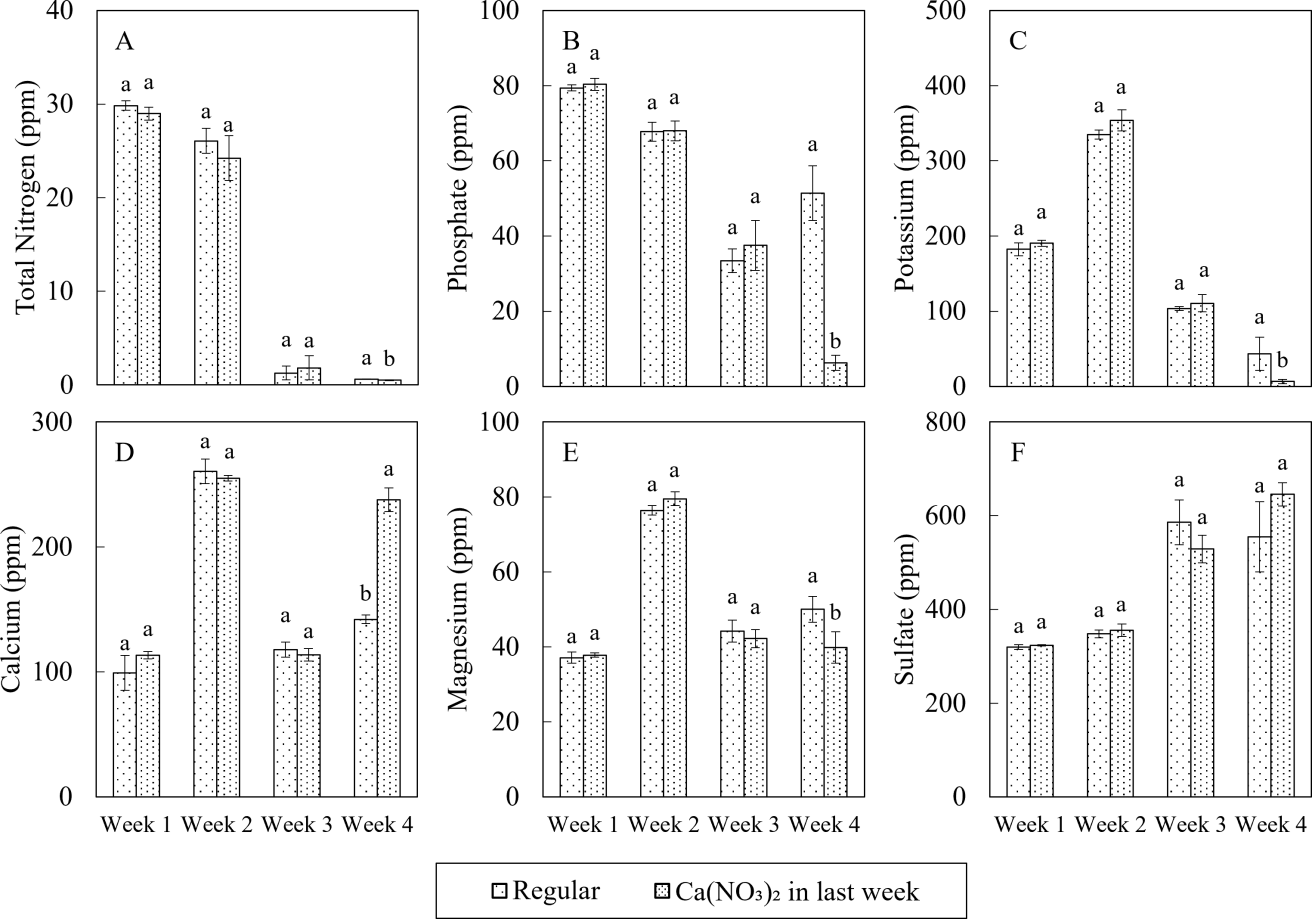
Effect of varying nutrient mixing protocols in the final week on macro nutrition concentrations including: total nitrogen **(A)**, phosphate **(B)**, potassium **(C)**, calcium **(D)**, magnesium **(E)**, and sulfur **(F)** in nutrient solution on the 1st, 2nd, 3rd, and 4th weeks after transplanting of kale in a nutrient-film technique hydroponic system containing nutrient solutions with EC of 1.8 dS·m^−1^. Data points with different letters are significantly different according to Tukey’s test (α = 0.05). Error bars represent the standard errors (n = 3).

Macro nutrient concentrations in nutrient solutions differed under two treatments in the final week of the study (**Figure 3**). Compared to the control, the nutrient solution in the Ca(NO_3_)_2_-only treatment had 19.1%, 87.8%, 84.9%, and 20.3% lower N, P, K and Mg, respectively, as well as 67.3% higher calcium. However, there was no significant difference in the concentration of sulfate. Greater calcium concentration in the Ca(NO3)2-only treatment resulted from increased application of calcium in that treatment. Interestingly, despite the extra nitrogen in the treatment, the total nitrogen concentration was still significantly lower than the control, aligning with the reduced concentrations of other macro nutrients. Compared to the original nutrient composition (**Table 1**), the macronutrient profile in the Ca(NO_3_)_2_-only treatment was significantly altered during the final week of production. N, P, and K levels decreased by 89.1%, 14.8%, and 8.1%, respectively, while Ca, Mg and S levels increased by 38.9%, 55.5% and 674.0%, respectively. These results may reflect higher nutrient uptake in the Ca(NO_3_)_2_-only treatment due to improved balance of nutrients accumulated in the stock tank.

### 3.2 Photosynthetic properties and water use efficiency

**Figure 4:**
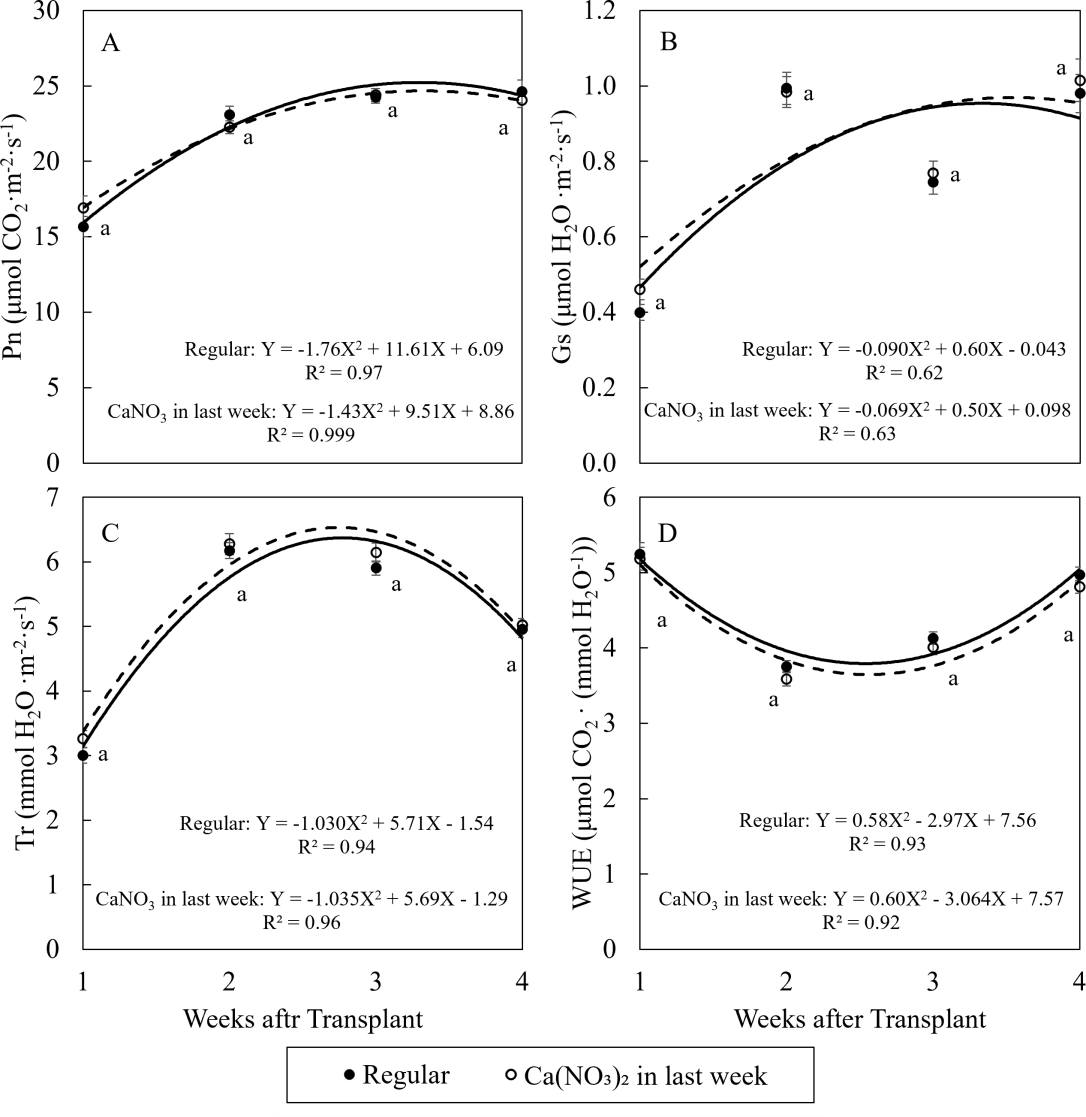
Effect of Ca(NO_3_)_2_ in the final week on the net photosynthetic rate **(A)**, stomata conductance **(B)**, transpiration rate **(C)**, and water use efficiency **(D)** of kale on the 1st, 2nd, 3rd, and 4th weeks after transplanting into a nutrient-film technique hydroponic system containing nutrient solutions with EC of 1.8 dS·m^−1^. Data points with different letters are significantly different according to Tukey’s test (α = 0.05). Error bars represent the standard error (n = 24).

The changing trend of net photosynthetic properties of kale were similar under two treatments during the study (**Figure 4**). In general, Pn and Gs increased over time, and Tr increased in the first two weeks then reduced, while WUE reduced in the first week then increased over time. These trends were aligned with higher plant growth rate and thicker cuticles over time. However, there was no significant difference in net photosynthetic properties for kale among treatments during the study.

**Figure 5:**
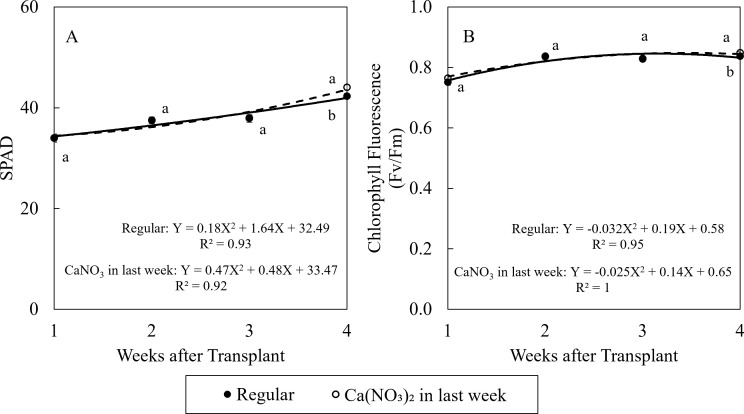
Effect of Ca(NO_3_)_2_ in the final week on relative chlorophyll content (SPAD) **(A)**, and chlorophyll fluorescence **(B)** of kale on the 1st, 2nd, 3rd, and 4th weeks after transplanting into a nutrient-film technique hydroponic system containing nutrient solutions with EC of 1.8 dS·m^−1^. Data points with different letters are significantly different according to Tukey’s test (α = 0.05). Error bars represent the standard error (n = 24).

Enhanced nitrogen supplementation in the final week improved kale growth (**Figure 5**). Compared to the control, treatment of Ca(NO_3_)_2_ had 4.2% higher SPAD value and 1.2% higher chlorophyll fluorescence value. After seedlings recovered from transplant shock, the SPAD values in kale were all higher than 0.83, indicating no stress in leaves in both treatments in this study (Björkman and Demmig, 1987).

### 3.3 Plant growth and yield

**Figure 6:**
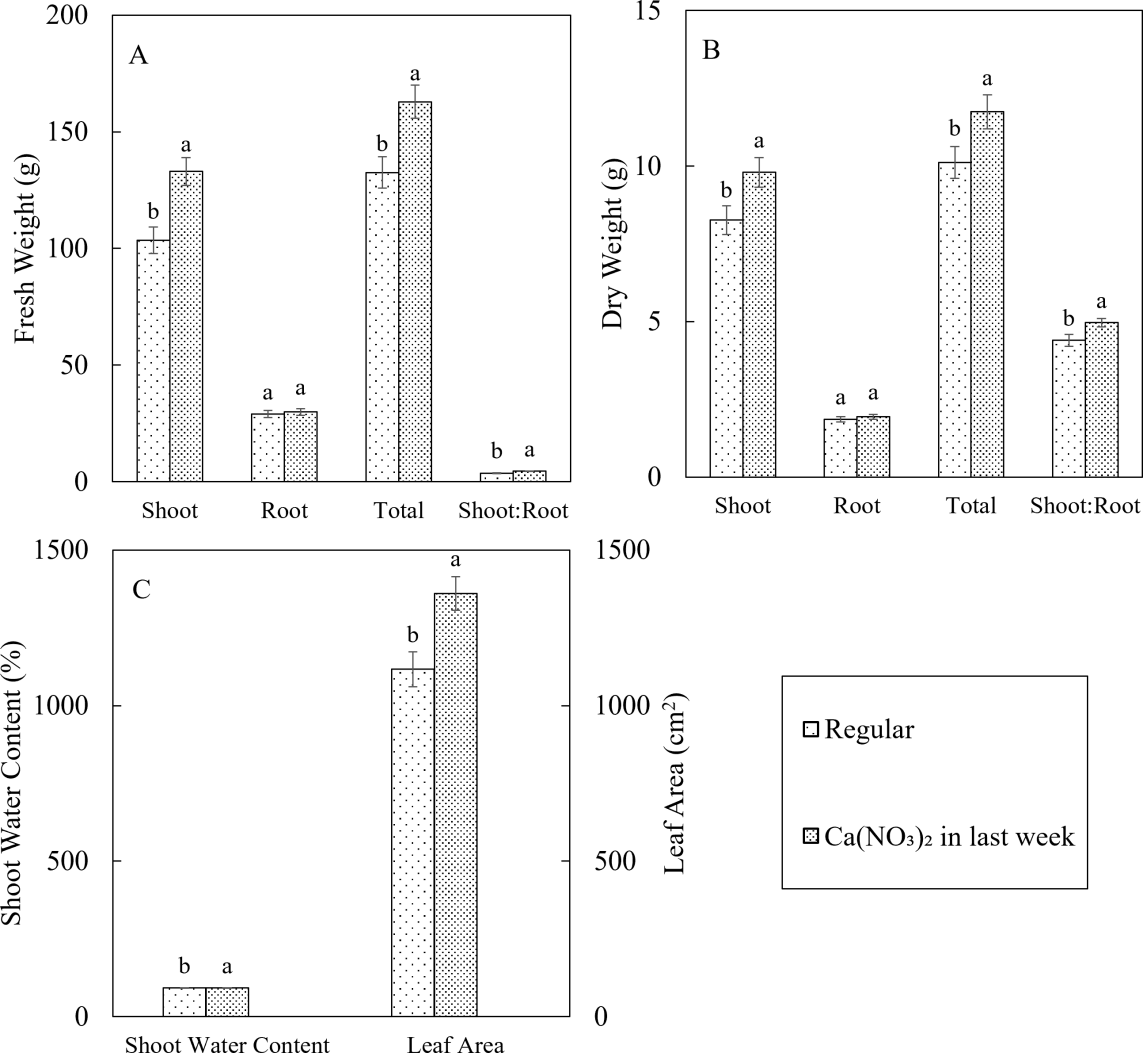
Effect of Ca(NO_3_)_2_ in the final week on plant tissue fresh weight **(A)**, dry weight **(B)**, shoot water content and leaf area **(C)** of kale in 4 weeks after transplanting into a nutrient-film technique hydroponic system containing nutrient solutions with electrical conductivity (EC) of 1.8 dS·m^−1^. Data points with different letters are significantly different according to Tukey’s test (α = 0.05). Error bars represent standard errors (n = 72).

Enhanced calcium and nitrogen supplementation in the final week significantly improved kale yields, shoot water content, and leaf area (**Figure 6**). Compared to the control, the Ca(NO_3_)_2_-only treatment resulted in a 28.5% increase in shoot fresh weight, a 22.9% increase in total fresh weight, and a 22.1% higher fresh shoot-to-root ratio. Additionally, the Ca(NO_3_)_2_-only treatment led to an 18.6% increase in shoot dry weight, a 4.8% increase in total dry weight, and a 13.0% higher dry shoot-to-root ratio. Furthermore, shoot water content was 0.64% higher, and leaf area increased by 21.8% under the Ca(NO_3_)_2_-only treatment. These findings are consistent with the enhanced plant growth rate observed with increased nitrogen and calcium supplementation. Importantly, there were no significant differences in root biomass between treatments, indicating that the reduction in other nutrient elements in the Ca(NO_3_)_2_-only treatment did not negatively impact root growth and development.

### 3.4 Plant tissue macro and micronutrient concentrations

**Figure 7:**
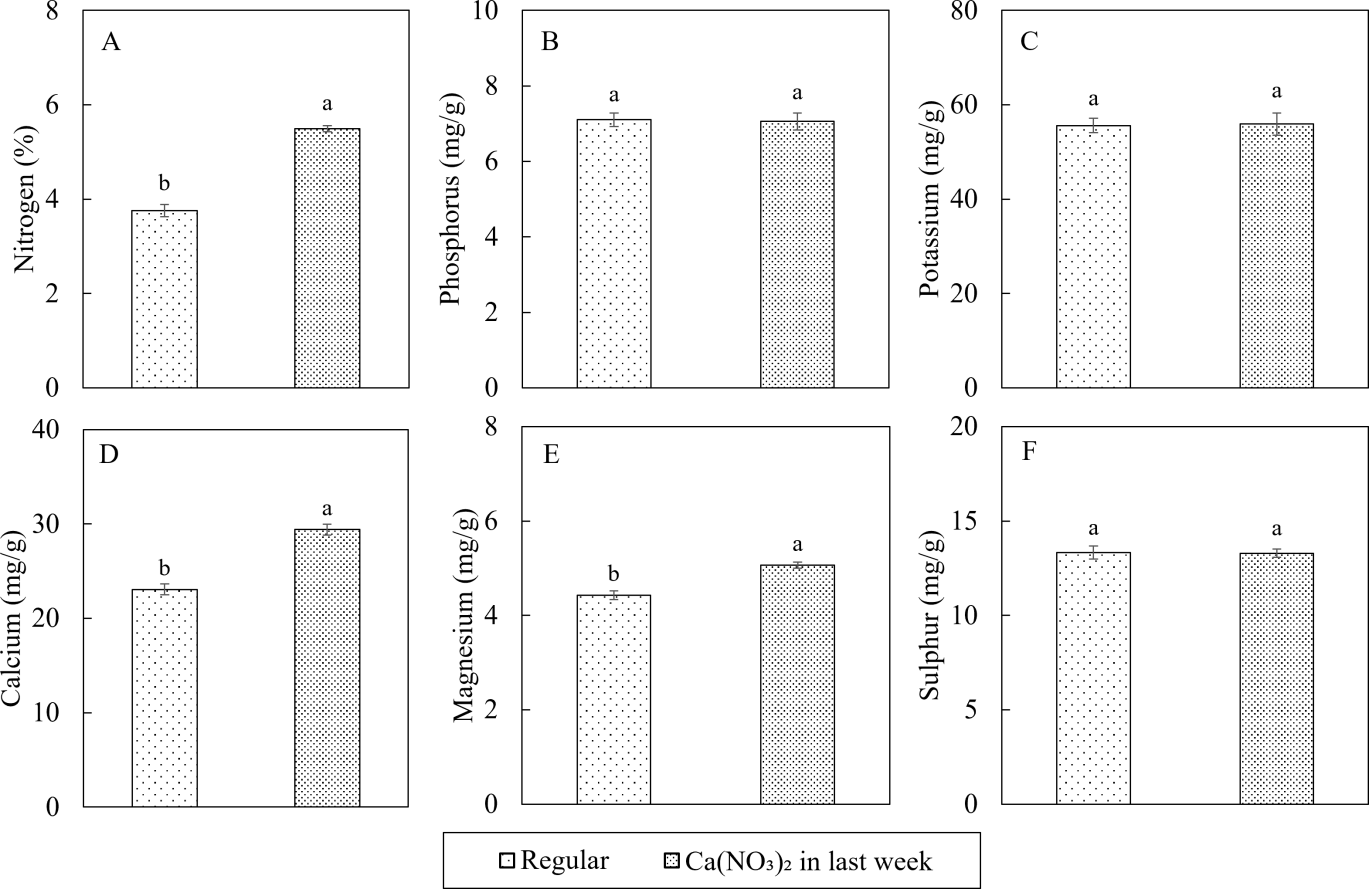
Effect of Ca(NO_3_)_2_ in the final week on macro nutrition concentrations in the shoot part of kale including: nitrogen **(A)**, phosphorus **(B)**, potassium **(C)**, calcium **(D)**, magnesium **(E)**, and sulfur **(F)** at 4 weeks after transplanting into a nutrient-film technique hydroponic system containing nutrient solutions with EC of 1.8 dS·m^−1^. Data points with different letters are significantly different according to Tukey’s test (α = 0.05). Error bars represent the standard errors (n = 18).

Macro and micronutrient concentrations in the tissues were within or above the sufficiency range based on Bryson et al. (2014) and Kopsell et al. (2005). In terms of the plant tissue macro nutrient concentrations, additional application of calcium nitrate improved not only the nitrogen and calcium concentrations in kale shoot, but also led to increased magnesium concentration (**Figure 7**). Compared to the control, the nutrient concentrations in the Ca(NO_3_)_2_-only treatment had 46.0%, 27.5%, and 14.4% higher total nitrogen (N), calcium (Ca), and magnesium (Mg), respectively. All other nutrient concentrations were not affected by treatment. The accumulation of Mg in the treatment could be the result of synergistic effect between N and Mg (Mulder, 1956).

**Figure 8:**
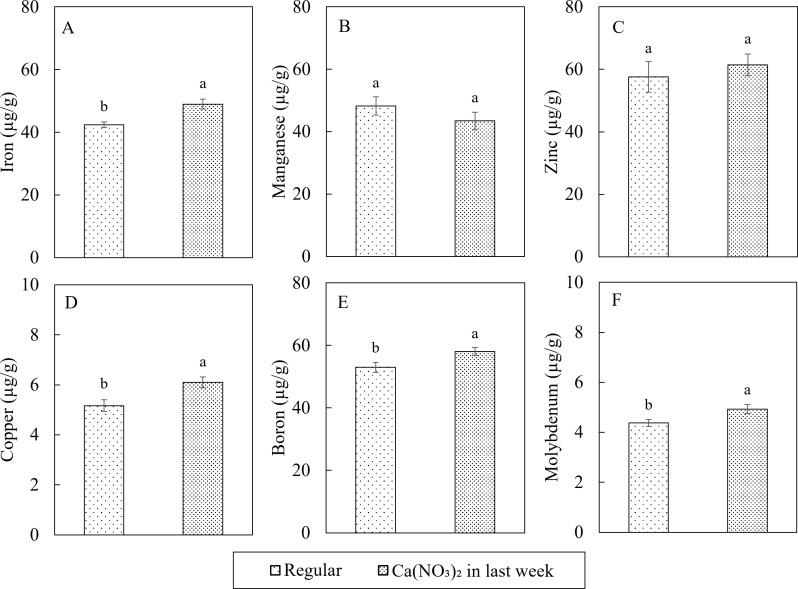
Effect of Ca(NO_3_)_2_ in the final week on micro nutrition concentrations in the shoot part of kale of iron **(A)**, manganese **(B)**, zinc **(C)**, copper **(D)**, boron **(E)** and molybdenum **(F)** at 4 weeks after transplanting into a nutrient-film technique hydroponic system containing nutrient solutions with EC of 1.8 dS·m^−1^. Data points with different letters are significantly different according to Tukey’s test (α = 0.05). Error bars represent the standard errors (n = 18).

In terms of the plant tissue micronutrient concentrations, extra application of calcium nitrate improved the iron (Fe), copper (Cu), boron (B) and molybdenum (Mo) concentrations in kale shoot (**Figure 8**). Compared to the control, the nutrient concentrations in the Ca(NO_3_)_2_-only treatment had 15.4%, 18.2%, 9.5%, and 12.6% higher Fe, Cu, B and Mo, respectively. However, manganese (Mn) and zinc (Zn) concentrations were not affected by the treatment.

### 3.5 Plant tissue nitrate and phytochemicals concentrations

**Figure 9:**
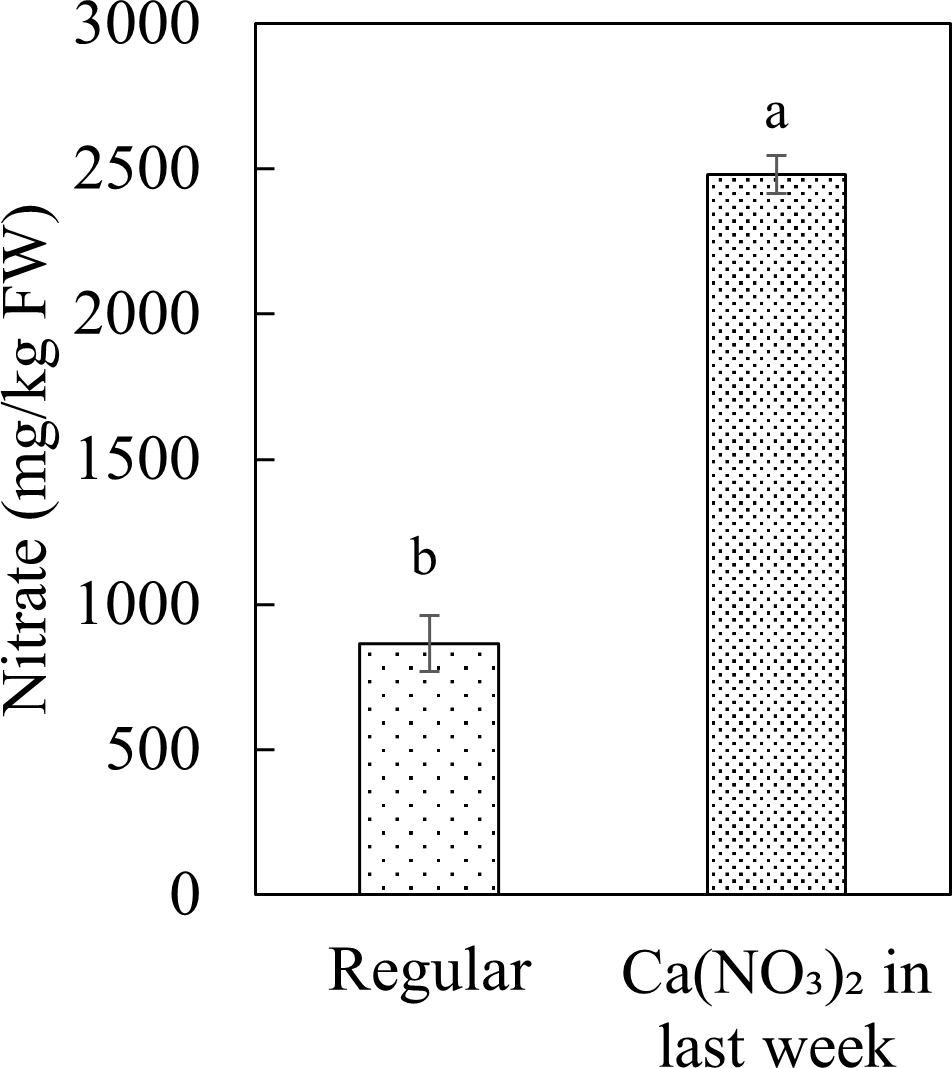
Effect of Ca(NO_3_)_2_ in the final week on the nitrate concentration in the shoot part of kale at 4 weeks after transplanting into a nutrient-film technique hydroponic system containing nutrient solutions with EC of 1.8 dS·m^−1^. Data points with different letters are significantly different according to Tukey’s test (α = 0.05). Error bars represent the standard errors (n=18).

Enhanced nitrogen supplementation in the final week increased kale tissue nitrate concentration (**Figure 9**). Kale is classified as a medium-nitrate content vegetable products (Colla et al., 2018). Nitrate concentration of kale in the Ca(NO_3_)_2_-only treatment in this study were comparable with previous research (EFSA, 2009; Erdoğan and Onar, 2012). The kale shoot nitrate concentration in the Ca(NO_3_)_2_-only treatment (2480.59 mg·kg^−1^) was 186.8% greater than the control (864.97 mg·kg^−1^). According to the report of European Food Safety Authority (EFSA), the maximum nitrate range for kale is 6,000-7,000 mg·kg^−1^ FW (European Union, 2011), indicating that the observed nitrate concentrations were within safe limits even with nitrogen enhancement.

**Figure 10:**
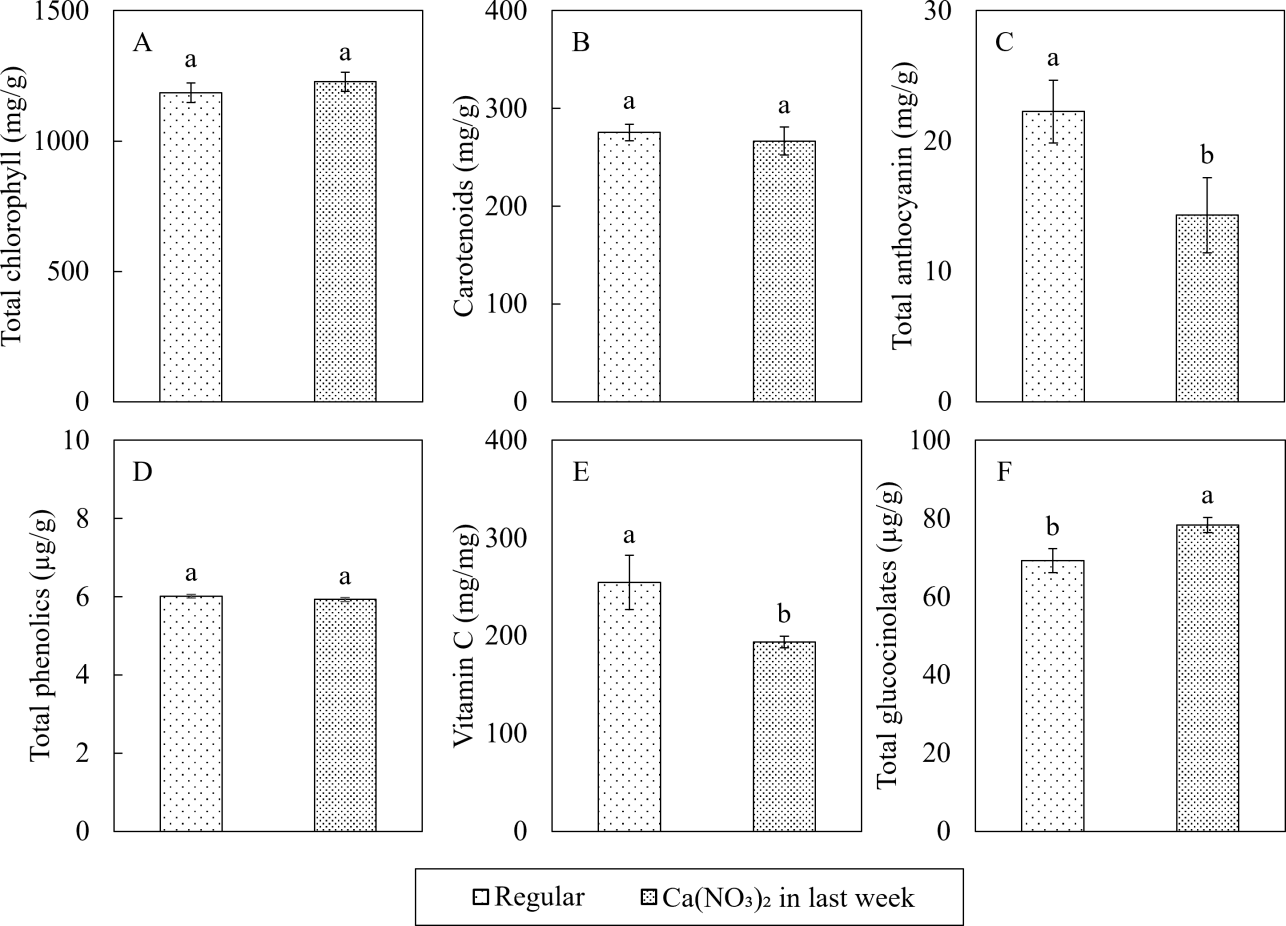
Effect of Ca(NO_3_)_2_ in the final week on the phytochemical concentrations of total chlorophyll **(A)**, carotenoids **(B)**, total anthocyanin **(C)**, total phenols **(D)**, vitamin C **(E)**, and total glucocinolates **(F)** in the shoot part of kale at 4 weeks after transplanting into a nutrient-film technique hydroponic system containing nutrient solutions with EC of 1.8 dS·m^−1^. Data points with different letters are significantly different according to Tukey’s test (α = 0.05). Error bars represent the standard errors (n = 24).

Enhanced nitrogen supplementation while reducing the remaining macro and micronutrients in the final week also affected some key phytochemicals concentrations in kale shoots (**Figure 10**). Compared to the control, the phytochemical concentrations in the Ca(NO_3_)_2_-only treatment had 35.7% and 24.0% lower total anthocyanin and vitamin C, but 13.2% higher total glucocinolates. However, all other phytochemicals (total chlorophyll, carotenoids, and total phenolics) were not affected by treatment.

## 4 Discussion

### 4.1 Changing mixing protocol in the final week of growth significantly improves plant growth, yield, and nutrient concentration

The modified nutrient adjustment protocol with application of Ca(NO_3_)_2_-only in the final week of kale production enhanced nutrient uptake, as evident by decreased concentrations of N, P, K, and Mg in the nutrient solution and increased accumulation of N, Ca, Mg, Fe, Cu, B, and Mo in kale shoots at harvest. Previous research on kale and collard indicated macronutrients other than N accumulate in the nutrient solution several-fold relative to original concentration in hydroponic systems during crop cycle (Yang et al., 2024). The modified mixing protocol adapted in this study mitigates the excess accumulation by reducing the input of these elements during the final phase of growth. Consequently, this balanced nutrient formulation coupled with supplemental Ca and N improved nutrient absorption during the peak growth phase of kale in this modified protocol. The observed increase in Mg accumulation could be attributed to a synergistic interaction between N and Mg (Mulder, 1956). Similarly, Kullmann et al. (1989) reported increased leaf K and Mg concentrations with rising N levels (up to 100 ppm N) in hydroponics-grown oilseed rape (*Brassica napus* L.), while Ca/Mg ratios and P concentrations remained stable, which aligned with our results.

Despite the additional N application, the total N concentration in the nutrient solution remained significantly lower than that in the control, indicating increased N uptake. This could explain the higher shoot nitrate concentration, which aligns with classification of kale as a medium-nitrate content vegetable (Colla et al., 2018). The nitrate concentration in Ca(NO_3_)_2_-only treated kale (2,480.6 mg·kg^-^¹ FW) remained well below the European Union’s safety threshold of 6,000-7,000 mg·kg^-^¹ FW (European Union, 2011). Therefore, changing the mixing protocol with Ca(NO_3_)_2_ could be a viable strategy for increasing yield in crops with a high nitrogen demand in hydroponic production (Yang et al., 2024).

The Ca(NO_3_)_2_-only treatment significantly improved shoot biomass and increased the shoot-to-root ratio, which positively correlated with higher nutrient uptake rates. This indicates that N and Ca availability were limiting factors for shoot growth, whereas root growth did not require additional nutrient supplementation. de Almeida et al. (2020) reported that N and Ca deficiencies manifest earlier than K and Mg deficiencies in hydroponically grown broccoli. Similarly, Burns (1994a) found that cabbage (cv. Stonehead) struggled to adapt to N deficiency due to limited N translocation to the roots, suggesting a minimum nitrate reserve of 0.1 mmol·g^-^¹ (6,200 mg·kg^-^¹) in shoots (Burns, 1994b. Our findings also align with Bruns et al. (1990), who reported enhanced shoot growth under increased N application (185–740 mg N·pot^-^¹) in hydroponically grown oilseed rape.

### 4.2 Changing mixing protocol in the final week of growth had no impact on acid and water usage, photosynthetic properties, or root growth

The Ca(NO_3_)_2_-only treatment did not affect acid and water usage or root growth. Nitrate uptake is known to increase pH, while calcium uptake reduces pH (Admin, 2017). Our results indicate a balanced uptake rate between nitrate and calcium, which likely explains the comparable acid usage for pH maintenance between treatments. These findings align with those of Chowdhury et al. (2024), who reported that NO_3_^-^-based nutrition (Ca(NO_3_)_2_) provides better pH buffering capacity than NH_4_^+^-based nutrition ((NH_4_)_2_SO_4_) in hydroponic systems for rapeseed (*Brassica napus* L.). This pH stability may be linked to the regulation of nutrient transport channel proteins.

Interestingly, root growth was not affected by the treatment, which may be attributed to the spatial limitations of the NFT system channels. Wang et al. (2017) demonstrated that root system architecture, particularly root size, plays a critical role in nitrogen use efficiency (NUE) and dry biomass accumulation in rapeseed. Similarly, Louvieaux et al. (2020) investigated the effects of nitrate supply (0.2 mM NO_3_^-^ [0.1 mM Ca(NO_3_)_2_ + 2.4 mM CaCl_2_], 5.0 mM NO_3_^-^ [2.5 mM Ca(NO_3_)_2_]) on biomass production and root morphology in oilseed rape and found that higher nitrate levels increased shoot and total biomass while decreasing the root-to-shoot biomass ratio indicating no impact root biomass. These results suggest that future studies should explore the optimal shoot-to-root ratio for maximizing both crop productivity and quality in hydroponic systems.

Furthermore, Ca(NO_3_)_2_-only treatment had no significant effect on photosynthetic properties, despite a slight increase in SPAD values and chlorophyll fluorescence. However, all measured parameters remained within the healthy range for kale growth, suggesting that the control treatment still provided suboptimal but sufficient growing conditions without negatively affecting plant health. Previous studies have explored the effects of optimized N/K ratios (Hu et al., 2019) and N/Mg ratios (Mao et al., 2022) on photosynthetic and growth characteristics of hydroponic grown kale. Future research should further investigate these nutrient interactions to refine nutrient management strategies in controlled environment agriculture.

### 4.3 Changing mixing protocol in the final week of growth affected leaf phytochemical concentration

The Ca(NO_3_)_2_-only treatment significantly reduced total anthocyanin and vitamin C concentration, raised total glucosinolate levels, but had no effect on chlorophylls and carotenoids in kale shoots.

A decline in antioxidant pigments after a nitrogen pulse is consistent with earlier reports. Shen et al. (2022) reported that anthocyanins and rutin in rapeseed play key roles in enhancing root cell resistance to oxidative damage and protecting against soil pathogen infections. Our findings suggest that modifying mixing protocol optimized growing conditions for kale compared to the control. Our previous research also indicated growth promotion via increased nutrient concentration can lead to reduction of some phytochemicals such as total anthocyanin, total phenols and vitamin C (Yang et al., 2021; Yang et al., 2024), suggesting that better nutrient supply can optimize the plant growth, update reducing the need for such defensive compounds.

By contrast, total glucocinolates responded positively. The concentrations we measured (69–78 µg g^-^¹ FW) fall within the typical range for kale and other *Brassica* greens (Sikorska-Zimny and Beneduce, 2021). Because glucocinolates contain both nitrogen and sulfur (Baenas et al., 2020), their synthesis is highly sensitive to the tissue N:S balance (Schonhof et al., 2007). Short−term enrichment with Ca(NO_3_)_2_ increased the external nitrate while effectively lowering available sulfate, creating an N−rich/S−poor environment known to raise both aliphatic and indole glucocinolates, even when total sulfur is adequate (del Carmen Martínez-Ballesta et al., 2013). Similar nitrate−induced glucocinolates gains have been documented in broccoli (Schonhof et al., 2007), kale (Groenbaek et al., 2016) and turnip (*Brassica rapa* spp. rapa) (Bonnema et al., 2019).

These results suggest that a brief late−cycle nitrogen boost can enrich kale with health−promoting glucosinolates without pushing nitrate levels beyond regulatory thresholds. Future work should refine N and S dosing schedules and test additional cultivars to optimize both yield and phytochemical quality in *Brassicaceae* family.

Although chlorophyll and carotenoid concentrations did not differ between treatments, many studies have generally observed increased chlorophyll and carotenoid concentrations (Chen et al., 2012; Kopsell et al., 2017; Ogunlela et al., 1989), as well as higher lutein and β-carotene levels (Kopsell et al., 2007), in response to appropriate nitrogen supplementation. These discrepancies may be due to differences in cultivar responses to nutrient treatments, as highlighted by Schulte auf’m Erley et al. (2017). This suggests the need for further screening of glucosinolate-rich cultivars and other accessions to optimize nutrient management strategies for phytochemical enhancement in hydroponically grown kale.

### 4.4 Practical applications for EC based hydroponic production

In EC-based nutrient management hydroponic systems, nutrient concentration is typically adjusted by modifying electrical conductivity (EC), often without tailoring individual nutrient concentrations. As a result, standard nutrient solutions deliver all macro- and micronutrients continuously throughout the crop cycle, regardless of changing plant demand. This can lead to overaccumulation of certain nutrients and depletion of others, particularly toward the end of production, resulting in nutrient imbalances that limit uptake efficiency and reduce crop performance (Yang et al., 2021 & Yang et al., 2024; Samarakoon et al., 2020).

Analysis of the original solution in this study revealed considerable nutrient imbalances during the final week of production. While N was nearly depleted due to high plant uptake, elements such as Ca, Mg and S accumulated excessively in the solution. This overaccumulation not only results in fertilizer wastage but may also inhibit the uptake of other essential nutrients due to ionic competition and osmotic stress (Ye et al., 2022; Dubey et al., 2021). In contrast, the Ca(NO_3_)_2_-only treatment applied during the final week provided a more balanced and crop-specific nutrient composition. Compared to the original solution, N levels decreased by 89.1%, directly addressing the plant’s need for targeted nitrogen resupply, while P and K—already abundant—decreased by 14.8% and 8.1%, respectively. Notably, Ca and Mg, which are essential for cell wall structure (Weng et al., 2022) and chlorophyll synthesis (Dukic et al., 2023), increased by 38.9% and 55.5%, respectively. However, increase in sulfur (674.0%) highlights the need for further optimization of nutrient formulation and solution management strategies, by adjusting the type of acid used for pH regulation. Overall, this revised mixing protocol better support plant demand during the final growth stage, optimizing nutrient use and minimizing excessive accumulation of less-needed elements. Furthermore, if nutrient solution is discarded at the end of production, there will be less nutrients in the drainage, minimizing fertilizer wastage, and environmental pollution.

From an economic standpoint, this modified strategy also reduces costs. The price for Hydro Grow Leafy Green Fertilizer ranges from $3.8 to $12.0 per pound (equivalent to $0.80-$2.54 per liter of stock solution), whereas the cost for Ca(NO_3_)_2_ is less with prices ranging between $0.56 and $6.25 per pound ($0.091-$1.02 per liter of stock solution). By adopting a Ca(NO_3_)_2_-only fertigation protocol in the final week of production, growers can reduce fertilizer expenses by 16.5% to 40.9%. Morever, this approach can lead to a 28% increase in kale yield, offering a cost-effective and productivity-enhancing strategy for hydroponic production.

## 5 Conclusion

For EC based nutrient management of hydroponics, use of only Ca(NO_3_)_2_ during the final week of kale production significantly enhanced nutrient uptake, leading to improved plant growth and increased yield. Higher nitrogen and calcium availability promoted greater nutrient assimilation, particularly for N, Ca, Mg, and trace elements such as Fe, Cu, B, and Mo.

However, phytochemical analysis revealed a reduction in anthocyanin and vitamin C concentration, while glucosinolate levels increased slightly. These findings highlight the complex interplay between nutrient supplementation and secondary metabolite synthesis, suggesting potential tradeoffs between yield and certain qualitative attributes.

Overall, the current study introduces a novel nitrogen and calcium supplementation method for EC based hydroponic crop management that can enhance kale production in hydroponic systems leading to increased yield, reduced fertilizer costs, and increased resource use efficiency. Future research should focus on using similar nutrient management strategies to other hydroponic leafy greens for yield improvements by balancing nutritional composition in the nutrient solution.

## Data Availability

The raw data supporting the conclusions of this article will be made available by the authors, without undue reservation.
